# Microbiota of Palm Oil Mill Wastewater in Malaysia

**DOI:** 10.21315/tlsr2018.29.2.10

**Published:** 2018-07-06

**Authors:** Jeremiah David Bala, Japareng Lalung, Adel Ali Saeed Al-Gheethi, Kaizar Hossain, Norli Ismail

**Affiliations:** 1 Environmental Technology Division, School of Industrial Technology, Universiti Sains Malaysia, 11800 USM Pulau Pinang, Malaysia; 2 Micro-pollution Research Centre (MPRC), Department of Water and Environmental Engineering, Faculty of Civil & Environmental Engineering, Universiti Tun Hussein Onn Malaysia, 86400 Batu Pahat, Johor, Malaysia

**Keywords:** Biodegradation, Industry, Malaysia, MALPOM Sdn. Bhd., Microbiota, Palm Oil Mill Effluent (POME), Wastewater, Biodegradasi, Industri, Malaysia, MALPOM Sdn. Bhd., Microbiota, Efluen Kilang Minyak Sawit (POME), Sisa Air

## Abstract

This study was aimed at identifying indigenous microorganisms from palm oil mill effluent (POME) and to ascertain the microbial load. Isolation and identification of indigenous microorganisms was subjected to standard microbiological methods and sequencing of the 16S rRNA and 18S rRNA genes. Sequencing of the 16S rRNA and 18S rRNA genes for the microbial strains signifies that they were known as *Micrococcus luteus* 101PB, *Stenotrophomonas maltophilia* 102PB, *Bacillus cereus* 103PB, *Providencia vermicola* 104PB, *Klebsiella pneumoniae* 105PB, *Bacillus subtilis* 106PB, *Aspergillus fumigatus* 107PF, *Aspergillus nomius* 108PF, *Aspergillus niger* 109PF and *Meyerozyma guilliermondii* 110PF. Results revealed that the population of total heterotrophic bacteria (THB) ranged from 9.5 × 10^5^ – 7.9 × 10^6^ cfu/mL. The total heterotrophic fungi (THF) ranged from 2.1 × 10^4^ – 6.4 × 10^4^ cfu/mL. Total viable heterotrophic indigenous microbial population on CMC agar ranged from 8.2 × 10^5^ – 9.1 × 10^6^ cfu/mL and 1.4 × 10^3^ – 3.4 × 10^3^ cfu/mL for bacteria and fungi respectively. The microbial population of oil degrading bacteria (ODB) ranged from 6.4 × 10^5^ – 4.8 × 10^6^ cfu/mL and the oil degrading fungi (ODF) ranged from 2.8 × 10^3^ – 4.7 × 10^4^ cfu/mL. The findings revealed that microorganisms flourish well in POME. Therefore, this denotes that isolating native microorganisms from POME is imperative for effectual bioremediation, biotreatment and biodegradation of industrial wastewaters.

## Abstract (ms)

Kajian ini bertujuan untuk mengenal pasti mikroorganisma asli daripada efluen kilang minyak sawit (POME) dan untuk menentukan beban mikrob. Pengasingan dan pengenalpastian mikroorganisma asli tertakluk kepada kaedah piawai mikrobiologi dan penjujukan gen 16S rRNA dan 18S rRNA. Penjujukan daripada gen16S rRNA dan 18S rRNA untuk strain mikrob menunjukkan bahawa mereka dikenali sebagai *Micrococcus luteus* 101PB, *Stenotrophomonas maltophilia* 102PB, *Bacillus cereus* 103PB, *Providencia vermicola* 104PB, *Klebsiella pneumonia* 105PB, *Bacillus subtilis* 106PB, *Aspergillus fumigates* 107PF, *Aspergillus nomius* 108PF, *Aspergillus niger* 109PF dan *Meyerozyma guilliermondii* 110PF. Keputusan menunjukkan bahawa jumlah populasi bakteria heterotropik (THB) berkisar antara 9.5 × 10^5^ – 7.9 × 10^6^ cfu/mL. Jumlah heterotopic fungi (THF) berkisar antara 2.1 × 10^4^ – 6.4 × 10^4^ cfu/mL. Jumlah populasi mikrob asli heterotropik pada medium agar CMC masing-masing berkisar antara 8.2 × 10^5^ – 9.1 × 10^6^ cfu/mL (bakteria) dan 1.4 × 10^3^ – 3.4 × 10^3^ cfu/mL (fungi). Penemuan mendapati bahawa mikroorganisma berkembang dengan baik di dalam efluen kilang minyak kelapa sawit (POME). Oleh itu, ini menunjukkan bahawa pengasingan mikroorganisma asli dari efluen kilang minyak sawit (POME) adalah penting untuk bioremediasi, rawatan bio dan biodegradasi sisa industri yang berkesan.

## INTRODUCTION

Industrial wastewaters are essential habitat for diverse microbes. Generally, some of the microorganisms have been used for biotreatment of wastewaters (Abdel-Raouf *et al.* 2012; Bala *et al.* 2014a, 2014b, 2014c; Bala *et al.* 2015a, 2015b; Bala 2016). Microorganisms domiciled in diverse wastewaters can also cause diseases such as tuberculosis, cholera, typhoid, dermatomycosis, hepatitis and dysentery (Shaaban *et al.* 2004).

Palm oil industry has become one of the most important agricultural based industries in Malaysia that produce colossal amount of oily liquid wastewater universally named as palm oil mill effluent (POME) (Ahmad *et al.* 2005; Rupani *et al.* 2010; Mohammed *et al*. 2014). Palm oil mill wastewater is produced during oil extraction processes in palm oil mill industries. POME is an extremely polluting wastewater that contaminates the environment when released directly into rivers, streams or lakes devoid of treatment.

POME; in addition include large amounts of solids, both suspended solids and total dissolved solids in the range of 18,000 mg/L and 40,500 mg/L correspondingly. These solids are usually named palm oil mill sludges (POMS). The solid waste that are formed in the process of extraction are the leaves, trunk, decanter cake, empty fruit bunches, seed shells and fibre from the mesocarp (Rupani *et al.* 2010).

Raw POME is a warm, acidic (pH between 4 and 5), brownish colloidal suspension having lofty concentrations of organic matter, elevated amounts of total solids (40,500 mg/L), oil and grease (4,000 mg/L), chemical oxygen demand (COD) (50,000 mg/L) and biochemical oxygen demand (BOD) (25,000 mg/L) (Ma 2000). The wastewater from palm oil mill can cause significant ecological problems, if released untreated (Singh *et al.* 2010). The chemical oxygen demand (COD) and biochemical oxygen demand (BOD) values of palm oil mill wastewater are high enough to cause serious pollution and environmental problem to the rivers. Chemical oxygen demand and biochemical oxygen demand of palm oil mill wastewater are very high and COD values greater than 60,000 mg/L are often reported (Bala *et al.* 2015a; Bala 2016). Accordingly, the adverse environmental impact from the palm oil industry cannot be overlooked. Consequently, the challenge of converting POME into an environmental friendly waste necessitates a well-organised treatment and effectual removal method.

The physicochemical properties of POME are well documented. Conversely, the microbiological aspect is overlooked; as such there seem to be dearth of information on the microbiota been documented proving that a well-developed understanding of these is needed. Therefore, this study represents one of the few studies in Malaysia. The diverse microbiota communities are known to participate effectively in the biodegradation and bioremediation of POME. Consequently, the study on the microbiological characteristics of POME lays a basis to promote better understanding of the types and nature of microorganisms domicile in POME. This will provide evidence of the microbiota characteristics of POME. Their involvement in biodegradation and biotreatment of POME may possibly abet in achieving higher reduction of organic load present in POME. This study was designed to explore the microorganisms associated with palm oil mill wastewater and to establish the microbial load from MALPOM Sdn. Bhd. in Pulau Pinang, Malaysia.

## MATERIALS AND METHODS

### Sample Collection and Preservation

Raw POME was collected aseptically from MALPOM Sdn. Bhd., Pulau Pinang, palm oil mill industry in a sterile microbiological container (20 L) and brought back to the laboratory. In collecting raw POME sample from the POME holding tank, the mouth of the tap connected to the holding tank was swabbed with cotton wool soaked in ethanol. This was done in order to disinfect the mouth of the tap. The tap was allowed to run for few minutes and the container was used to collect the POME sample and quickly corked. Prior to sample collection, the POME sample inside the container was inverted a few times in ordered to rinse the inside wall of the container. The sample was later poured out into the surrounding. This step was done three times and the container was finally placed to collect the POME sample. The POME sample was kept in an ice box while transporting to School of Industrial Technology laboratory, Universiti Sains Malaysia and preserved at 4°C until further experiment in order to stop the wastewater from undergoing biodegradation due to microbial action (American Public Health Association [APHA] 2005). Sample was brought out from the refrigerator and left at room temperature before use.

### Isolation and Enumeration of Total Heterotrophic Indigenous Palm Oil-Utilising and Cellulose Utilising Bacteria From POME

The populations of microorganisms in the raw POME sample was enumerated using standard spread plate method (APHA 2005; Bala *et al.* 2015a; Bala 2016). The POME was well shaken to homogenised suspension and thereafter, ten-fold (10-fold) serial dilution was made by aseptically transferring one milliliter (1 mL) of the homogenised suspension into a sterile test tubes containing nine milliliter (9 mL) of sterile, distilled water. Then, using a sterile pipette, 0.1 mL aliquots of the dilutions were aseptically removed with a sterile pipette and separately spread plated with flamed-sterilised glass spreader (bent glass rod) on well-dried Nutrient Agar (NA), oil agar (Palm Oil Agar [POA]) Mineral Salts Medium (MSM) for bacteria and Carboxymethyl cellulose (CMC) agar plates for bacteria in triplicates for the enumeration of viable heterotrophic bacteria, palm oil utilising and cellulose utilising bacteria respectively. The plates were inoculated using spread plate technique (APHA 2005; Bala *et al.* 2015a; Bala 2016). The culture plates were incubated at 37°C for 24–48 h. Three uninoculated plates were used as control. After incubation, plates that contained 30–300 colony forming units (cfu) were selected and counted with the aid of a colony counter. Viable numbers of colonies on each plate were enumerated and expressed or recorded as colony forming units per milliliter (cfu/mL) of the sample. Colonies were purified by repeatedly subcultured aseptically on to fresh NA, oil agar and CMC agar and incubated at 37°C for 48 h to obtain discrete pure colonies. Pure colonies were then stored on NA, oil agar and CMC agar slants at 8°C to maintain viability for subsequent analysis and identification. Gram staining was performed for all the isolates. The medium was incorporated with Ketoconazole antifungal (known as funginox) to inhibit fungal growth.

### Preparation and Composition of Mineral Salt Medium (MSM) for Palm Oil Utilising Bacteria

The MSM (oil agar medium) for palm oil utilising bacteria was prepared according to the MSM composition of Zajic and Supplisson (1972). The composition of the medium was NH_4_Cl (4.0 g), K_2_HPO_4_ (1.8 g), KH_2_PO_4_ (1.2 g), MgSO_4_.7H_2_O (0.2 g), NaCl (0.1 g), FeSO_4_ (0.01 g), 15 g agar and distilled water, 1 L). The medium was used for isolation, enumeration and identification of palm oil-utilising bacteria (oil degraders). The medium was prepared by the addition of 1% (v/v) palm oil as sole source of carbon and energy, sterilized with 0.45 μm pore size Millipore filter paper to sterile MSM, which has been cooled to 45°C under aseptic condition. 200 mg ketoconazole antifungal (known as funginox) was added to prevent fungal growth. The MSM and palm oil were then mixed thoroughly and dispensed into sterile Petri dishes to solidify.

### Isolation and Enumeration of Total Heterotrophic Indigenous Palm Oil-Utilising and Cellulose Utilising Fungi from POME

The standard procedures for serial dilution aforementioned for bacterial isolation were followed for fungal isolation. Thereafter, using a sterile pipette, 0.1mL aliquots of the dilutions were aseptically removed with a sterile pipette and separately spread plated with flamed-sterilised glass spreader (bent glass rod) on well-dried Potato Dextrose Agar (PDA), oil agar (POA) MSM for fungi and Carboxymethyl cellulose (CMC) agar plates for fungi in triplicates for the enumeration of viable heterotrophic fungi, palm oil utilising and cellulose utilising fungi respectively. The plates were inoculated on the surface using the standard spread plate technique (APHA 2005). The plates were allowed to remain undisturbed for 25 min in the laminar flow before been inverted and incubated.

The culture plates were incubated at 28°C for 5–7 days (APHA 2005). Three uninoculated plates were used as control. After incubation, viable numbers of colonies on each plate were enumerated and expressed or recorded as colony forming unit per milliliter (cfu/mL). Colonies were purified by repeatedly sub culturing aseptically on to fresh PDA, oil agar and CMC agar and incubated at 28°C for 5–7 days to obtain discrete pure colonies. Pure colonies were then stored on PDA, oil agar and CMC agar slants at 8°C to maintain viability for subsequent analysis and identification. Staining was also performed for all the isolates using lacto phenol cotton blue solution. The medium was incorporated with Altacef antibiotic to inhibit bacterial growth.

### Preparation and Composition of Mineral Salt Medium (MSM) for Palm Oil Utilising Fungi

The MSM (oil agar medium) for palm oil utilising fungi was prepared according to the MSM composition of Mills *et al*. (1978) as modified by Okpokwasili and Okorie (1988).The composition of the medium was NaCl, 10.0g; MgSO_4_.7H_2_O, 0.42g; KCl, 0.29 g; KH_2_PO_4_, 0.83 g; Na_2_HPO_4_, 1.25 g; NaNO_3_, 0.42 g; agar, 20 g; distilled water, 1 L and pH of 7.2. The medium was used for isolation, enumeration and identification of palm oil-utilising fungi (oil degraders). The medium was prepared by the addition of 1% (v/v) palm oil as sole source of carbon and energy, sterilised with 0.45 μm pore size Millipore filter paper to sterile MSM, which has been cooled to 45°C under aseptic condition. 250 mg Altacef antibiotic, was added to prevent bacterial growth. The MSM and palm oil were then mixed thoroughly and dispensed into sterile Petri dishes to solidify.

### Identification of Bacteria Isolates by Sequencing of 16S rRNA Gene

Initial identification of individual bacterial isolates was achieved by standard tests (Bergey *et al.* 1994). Such identification included the shape of cells, Gram’s reaction and colony morphology on solid nutrient media. Genetic identification of bacterial isolates was performed by determining nucleotide sequences of 16S rRNA genes using commonly used primers (Table 1) for amplifying the DNA between positions 27 and 1492 of bacterial 16S rRNA genes. Genetic identification of the pure cultures of bacterial isolated from POME were sent to Centre for Chemical Biology (CCB), Universiti Sains Malaysia for sequencing of the 16S rRNA gene. Inoculum preparation was carried out by inoculating bacteria strains in nutrient broth, fungi in potato dextrose broth, incubated for 24 h (bacteria), 2–3 days (fungi) at 37°C and 28°C respectively.

### Identification of Fungal Isolates by Sequencing of 18S rRNA Gene

Initial identification of individual fungal isolates was based on microscopic staining of fungi using lactophenol blue solution (Lactophenol cotton blue solution) and macroscopic appearance which comprise pigmentation/colour, identified on the basis of cultural (colour and colonial appearance of fungal colony) and morphological characteristics in lacto-phenol blue solution wet mount by compound microscope. Genetic identification of fungal isolates was performed by determining nucleotide sequences of 18S rRNA genes using commonly used primers (Table 2) for amplifying the DNA. Genetic identification of the pure cultures of fungal isolated from POME were sent to CCB, Universiti Sains Malaysia for sequencing of the 18S rRNA gene.

## RESULTS AND DISCUSSION

### Microbial Populations of POME Sample

The microbial population, total heterotrophic bacteria (THB) and total heterotrophic fungi (THF) of POME are presented in Table 3 and oil degrading bacteria (ODB) and oil degrading fungi (ODF) are presented in Table 4.

The population of total heterotrophic bacteria (THB) ranged from 9.5 × 10^5^ – 7.9 × 10^6^ cfu/mL. The total heterotrophic fungi (THF) ranged from 2.1 × 10^4^– 6.4 × 10^4^ cfu/mL. Total viable heterotrophic indigenous (authochthonous) microbial population on CMC agar ranged from 8.2 × 10^5^ – 9.1 × 10^6^ cfu/mL and 1.4 × 10^3^– 3.4 × 10^3^ cfu/mL for bacteria and fungi respectively. The microbial population of oil degrading bacteria (ODB) ranged from 6.4 × 10^5^ – 4.8 × 10^6^ cfu/mL and the oil degrading fungi (ODF) ranged from 2.8 × 10^3^ – 4.7 × 10^4^ cfu/mL (Tables 3 and 4). The findings revealed that ODB and ODF flourish well in oily waste water. Awotoye *et al.* (2011) reported THB, THF, ODF and ODB population of 1.8 × 10^6^ cfu/g, 9.5 × 10^2^ cfu/g, 1.2 × 10^2^ cfu/g and 4.0 × 10^2^ cfu/g in that order at the point of POME release from oil palm milling machine. Ugoji (1997) specified that THB and THF are 1.3 × 10^6^cfu/mL and 1.0 × 10^3^ cfu/mL correspondingly in POME.

In a related study, Okwute and Isu (2007a; 2007b) have reported total aerobic bacterial populations of 9.6 × 10^8^ cfu/mL, 1.64 × 10^9^ cfu/mL and 1.07 × 10^9^ cfu/mL in POME samples. In addition, Okwute (2013) has also confirmed the population of THB, THF and ODB as 4.0 × 10^9^ cfu/mL, 2.6 × 10^3^ cfu/mL and 2.6 × 10^3^ cfu/mL in that order. The counts were also comparable to those described by Serikovna *et al.* (2013) with the index of 10^8^ cfu/mL, 10^7^ cfu/mL and 2 × 10^8^ cfu/mL as well as Wu *et al.* (2009) who revealed in their study the count of 6.65 × 10^6^ cfu/mL from oily wastewaters. Ohimain *et al.* (2012a) has also stated that the population of total heterotrophic bacteria (THB) ranged from 7.4 × 10^5^ – 2.0 × 10^6^ cfu/mL and total heterotrophic fungi (THF) ranged from 3.1 – 5.7 × 10^4^ cfu/mL while the oil degrading bacteria (ODB) ranged from 6.5 × 10^5^ – 2.0 × 10^6^ cfu/mL and the oil degrading fungi (ODF) ranged from 3.1 – 5.6 × 10^4^ cfu/mL in POME sample. Bala *et al.* (2012) has also reported similar counts from pharmaceutical wastewater. These corroborate the presence of diverse microorganisms in wastewaters (Bala 2016).

Results from the present study aforementioned confirmed some disparity in the microbial counts. The variations in the range of microbial populations are an indication of several reasons such as nutrient, minerals, temperature, oxygen level, acidity, volume of wastewater (Okereke *et al.* 2007), concentration of oil and grease and sugars in the POME. High population of bacteria in the POME may possibly be linked with contaminations from poor sanitation in the mills (Okechalu *et al.* 2011), and intermittent disinfection of the environment. Besides, it may also be due to the handling process and the existing environmental conditions in the mills. The presence and growth of viable bacteria and fungi in POME may possibly be associated with the fact that POME is rich in carbohydrates, proteins, nitrogenous compounds, lipids, minerals, cellulose, hemicelluloses and lignin (Hii *et al.* 2012).

The microbes isolated in the present study conceivably derive their nutrients from the aforementioned compounds in raw POME. The microbial species found in POME has the prospective to degrade carbon source present in the POME. Bala *et al.* (2014b) and Bala (2016) has reported that *Micrococcus luteus* 101PB, *Stenotrophomonas maltophilia* 102PB, *Bacillus cereus* 103PB and *Bacillus subtilis* 106PB showed high lipase activity on solid media indicating their ability for degrading lipid (oil) as carbon source and producing lipase enzyme. The types of organisms isolated in the present study were also identified as oil degrading microorganisms by Bharathi and Vasudevan (2001) and Rahman *et al.* (2002) because of their ability to hydrolyse lipid (oil). Biodegradation is connected with the capability of bacteria and fungi to grow on and degrade carbon sources in industrial wastewaters (Haimann 1995). The high organic matter in palm oil mill wastewater possibly will have played an essential role in the abundance of aerobic and facultative anaerobic microbial strains in the present study.

### Genetic Identification of Bacteria and Fungi Isolates in POME Sample

Tables 1 and 2 present the microorganisms isolated from POME based on 16S rRNA gene and 18S rRNA genes for bacteria and fungi respectively. Identification of isolates was performed by determining nucleotide sequences of 16S rRNA and 18S rRNA genes for bacteria and fungi in that order. The isolates were identified by sequences analysis of 16S rRNA and 18S rRNA genes. Sequencing of the 16S rRNA and 18S rRNA of the microbial strains suggest that they were known as *Micrococcus luteus* 101PB, *Stenotrophomonas maltophilia* 102PB, *Bacillus cereus* 103PB, *Providencia vermicola* 104PB, *Klebsiella pneumoniae* 105PB, *Bacillus subtilis* 106PB, *Aspergillus fumigatus* 107PF, *Aspergillus nomius* 108PF, *Aspergillus niger* 109PF and *Meyerozyma guilliermondii* 110PF. Plates and Figures showing identified bacteria and fungi in POME sample is presented in Appendix A to H.

The results from the present study revealed that the microbes isolated are comparable to those found in areas polluted with wastewaters (Abass *et al.* 2012; Soleimaninanadegani & Manshad 2014; Bala *et al.* 2015a) and crude oil or petroleum hydrocarbons (Okereke *et al.* 2007). Bala *et al.* (2012) had also reported the isolation of *Bacillus subtilis* from industrial wastewater. Conversely, *Micrococcus luteus* 101PB, *Stenotrophomonas maltophilia* 102PB, *Bacillus cereus* 103PB, *Bacillus subtilis* 106PB, *Aspergillus fumigatus* 107PF and *Aspergillus niger* 109PF are lipase and cellulase producing organisms isolated from the present study.

The development of spores makes POME microorganisms to be quiescent and highly resistant to lethal consequence of boiling, dry heating and ultra violet radiation from the sunlight (Okechalu *et al.* 2011). Palm oil mill wastewater is a possible habitat for lipolytic and cellulolytic bacteria and fungi since it is rich in nutrients such as lipids (oil) and cellulosic materials (Ohimain *et al.* 2012a; 2012b; Bala 2016).

Ohimain *et al.* (2012a) isolated lipase and cellulase producing *Bacillus* sp from POME collected from palm oil processing environment. Asikong (1994) identified *Aspergillus* sp. as fungal species linked with lipase and cellulase production. *Aspergillus* sp. is particularly reported to be good producers of cellulase and lipase. These enzymes are responsible for the breakdown of cellulose and oil in POME (Wong *et al.* 2008). *Aspergillus niger* and *Aspergillus fumigatus* have been well-known for their capability to survive in oily wastewater such as Palm oil mill wastewater due to the presence of nutrients such as lipids (oil). Fungi are particularly aerobic and can also grow under environmental strained conditions such as low pH and poor nutrient status. Lipase facilitates the hydrolysis of lipid causing succeeding breakdown into fatty acid and alcohol (Guehi *et al.* 2007; Ghosh *et al.* 1996). Other researchers have also isolated comparable microbes aforementioned above at 28°C-37°C from POME sample (Bhumibhamon *et al.* 2002; Ohimain *et al.* 2012a; 2012b; Okwute 2013; Soleimaninanadegani & Manshad 2014; Bala 2016).

The prevalence of these microbes (bacteria and fungi) in palm oil mill wastewater may perhaps be due to their capability to make use of oil and cellulose as their sole carbon source which has been formerly reported by Ojumu *et al.* (2005), Bala *et al.* (2014b), Bala *et al.* (2015b), and Bala (2016). The use of POME as a carbon source by these microorganisms has been reported by Wu *et al.* (2007), Sira *et al.* (2010) and Bala (2016). The presence of *Micrococcus luteus* 101PB, *Stenotrophomonas maltophilia* 102PB, *Bacillus cereus* 103PB, *Bacillus subtilis* 106PB, *Aspergillus fumigatus* 107PF and *Aspergillus niger* 109PF isolated from POME sample in the current study revealed that these microorganisms are capable of biodegradation of oily wastewaters as reported by other researchers (Ohimain *et al.* 2012a; 2012b; 2012c; Nwuche & Ogbonna 2011).

Microorganisms present in POME have been used for the treatment of wastewaters such as palm oil mill wastewater and olive oil mill wastewater for the reduction of COD (Oswal *et al.* 2002; Ohimain *et al.* 2012a; Kamal *et al.* 2011; Neoh *et al.* 2013; Nawawi *et al.* 2010; Ahmad *et al.* 2011; Bala *et al.* 2014c; Bala *et al*. 2015a; Bala 2016). During degradation process, oil and cellulose in POME are broken down by effective microbes which make use of the organic waste present in palm oil mill wastewater and degrades these organic matters into water and carbondioxide (Singh *et al.* 2010; Jameel & Olanrewaju 2011). *Aspergillus fumigatu*s 107PF, *Aspergillus niger* 109PF, *Micrococcus luteus* 101PB, *Stenotrophomonas maltophilia* 102PB, *Bacillus cereus* 103PB, and *Bacillus subtilis* 106PB have been isolated for POME with potential to degraded oil and cellulose (Bala *et al.* 2014b; Bala *et al.* 2015b; Bala 2016). The aforesaid microbes thus exhibited comparable biodegradation potential with published literatures. The oily habitat in palm oil mill wastewater possibly will make available a good environment for lipolytic microorganisms to grow due to the oil present in the wastewater which serves as carbon source. However, the present of these microbes in POME are useful in degrading contaminated pollutants in wastewaters such as crude oil (hydrocarbon) (Ohimain *et al.* 2012a; Soleimaninanadegani & Manshad 2014). Palm oil mill wastewater is inhabited by dissimilar types of microbes which plays a fundamental task in the biotreatment, bioremediation and biodegradation of oil-containing wastewaters (Hassen-Aboushiba *et al.* 2013; Tan *et al.* 2015).

Conversely, in view of the fact that most of the microbes domiciled in POME form spores, it facilitate their survival and continued existence in harsh or stressed normal conditions of palm oil mill wastewater such as absence of air or free oxygen (anaerobiosis), soaring concentration of oil and grease (Okechalu *et al.* 2011; Ugoji 1997), and acidity (Leslie-Grady *et al.* 1999; Breccari *et al.* 1996; Poh & Chong 2009; Ugoji 1997). This corroborates with the study of Bala *et al.* (2015a) who reported in their investigation a low pH of 4.74 from raw palm oil mill wastewater in Malaysia. Under anaerobic conditions, methane and carbon dioxide are produced (Ugoji 1997). The anaerobic microflora inhabitant of palm oil mill wastewater sludge may well be valuable for the manufacture of biohydrogen and biogas production by fermentation during treatment (Vijayaraghavan & Ahmad 2006; Atif *et al.* 2005; Ismail *et al.* 2010). Table 5 revealed cultural characteristics of bacteria isolated from palm oil mill wastewater while Table 6 revealed microscopic, macroscopic morphology and cultural characteristics of fungi isolated from palm oil mill wastewater.

## CONCLUSION

Results from the current study revealed the presence of diverse types of microorganisms domiciled in palm oil mill wastewater. This conclusion suggests that microorganisms thrive well in palm oil mill wastewater. The investigation provides insight on the exploitation of microbial strains in biotreatment of industrial agricultural based wastewaters such as palm oil mill wastewater. The diversity of microbial strains isolated from palm oil mill wastewater provides a basis to promote better understanding of the types and nature of microorganisms domicile in palm oil mill wastewater. This will provide evidence on the microbiota characteristics of palm oil mill wastewater. Conversely, this signifies the optimism for identification of native microbes from palm oil mill wastewater for biodegradation and bioremediation of industrial wastewaters. Study on metagenomic and transcriptomics characterisation is required for further identification of microbial strains diversity using Next-Generation Sequencing (NGS).

## Figures and Tables

**Table 1:** Genetic Identification of bacterial isolates in POME

	Bacteria
Nucleotide Sequences	16S rRNA gene
Sequences of Primers	**27F: 5’-AGAGTTTGATCMTGGCTCAG-3’**
	**1492R: 5’-GGGTTACCTTGTTACGACTT-3’**
Strains	*Micrococcus luteus* 101PB **(Accession No. AB539843.1)**
	*Stenotrophomonas maltophilia* 102PB **(Accession No. JQ 619623.1)**
	*Bacillus cereus* 103PB **(Accession No. JF 432000.1)**
	*Providencia vermicola* 104PB **(Accession No. KC775772.1)**
	*Klebsiella pneumoniae* 105PB **(Accession No. GU128173.1)**
	*Bacillus subtilis* 106PB **(Accession No. KF624694.1)**

**Table 2:** Genetic Identification of fungal isolates in POME

	Fungi
Nucleotide Sequences	18S rRNA genes
Sequences of Primers	**ITS1 F: 5’-TCCGTAGGTGAACCTGCGG -3’**
	**ITS4 R: 5’-TCCTCCGCTTATTGATATGC-3’**
Strains	*Aspergillus fumigatus* 107PF **(Accession No. EU664467.1)**
	*Aspergillus nomius* 108PF **(Accession No. DQ467991.1)**
	*Aspergillus niger* 109PF **(Accession No. KC119204.1)**
	*Meyerozyma guilliermondii* 110PF **(Accession No. JN183444.1)**

**Table 3:** Microbial populations of POME.

Media	Isolates	Total heterotrophic counts (THC)
Nutrient agar (NA)	Bacteria	9.5 × 10^5^ − 7.9 × 10^6^ cfu/mL
Potato Dextrose agar (PDA)	Fungi	2.1 × 10^4^ − 6.4 × 10^4^ cfu/mL
Carboxymethyl cellulose (CMC) agar	Bacteria	8.2 × 10^5^ − 9.1 × 10^6^ cfu/mL
Carboxymethyl cellulose (CMC) agar	Fungi	1.4 × 10^3^ − 3.4 × 10^3^ cfu/mL

**Table 4:** Oil degrading microbes of POME.

Media	Isolates	Counts (cfu/mL)
Oil agar (MSM) Palm oil agar (POA)	Bacteria	6.4 × 10^5^ – 4.8 × 10^6^
Oil agar (MSM) Palm oil agar (POA)	Fungi	2.8 × 10^3^ – 4.7 × 10^4^

**Table 5:** Cultural characteristics of bacteria isolated from POME.

Organisms	Characteristics	Gram’s reaction
*Micrococcus luteus* 101PB	Circular, pinhead colonies which are convex with entire margins. Colonies produces a bright yellow, nondiffusable pigment	Positive cocci
*Stenotrophomonas maltophilia* 102PB	circular, smooth, convex, moist and pigmented colonies	Negative rod
*Bacillus cereus* 103PB	Large, irregular, opaque colonies. Smooth and moist colonies, whitish to cream	Positive rod
*Providencia vermicola* 104PB	Colonies are circular, shining, slimy, convex, and opaque with a brownish centre. Brown pigment is produced, colouring the medium around the colonies. Colonies are smooth with entire edges.	Negative rod
*Klebsiella pneumoniae* 105PB	Distinctive yeasty odor and bacterial colonies have a viscous/mucoid appearance	Negative rod
*Bacillus subtilis* 106PB	Dry, flat, and irregular, with lobate margins; colonies round or irregular; surface dull; become thick and opaque; whitish	Positive rod

**Table 6:** Microscopic, macroscopic morphology and cultural characteristics of fungi isolated from POME.

Organisms	Type of organisms	Microscopic morphology	Macroscopic morphology
*Aspergillus fumigatus* 107PF	Filamentous mold	Presence of rough conidiophore, with uni/biseriate phialides whose vesicle is round with radiate head. Brownish sclerotia were also observed	Presence of blue-green to yellow coloration from surface
*Aspergillus nomius* 108PF	Filamentous mold	Presence of septate hyphae and colourless and rough conidiophores with swollen vesicles	A brownish colour with a creamy edge that appears golden in the reverse of the septate
*Aspergillus niger* 109PF	Filamentous mold	Presence of septate hyphae, long and smooth conidiophores, and long unbranded sporangiospores with large, round head	Brownish-black mycelium with dark spores and often appears golden on the reverse side
*Meyerozyma guilliermondii* 110PF	Yeast	Clusters of small blastospores along the pseudohyphae and particularly at septal points. Pseudohyphae are short and few in number	Colonies are flat, moist, smooth, and cream to whitish in colour

**Plate 15:**
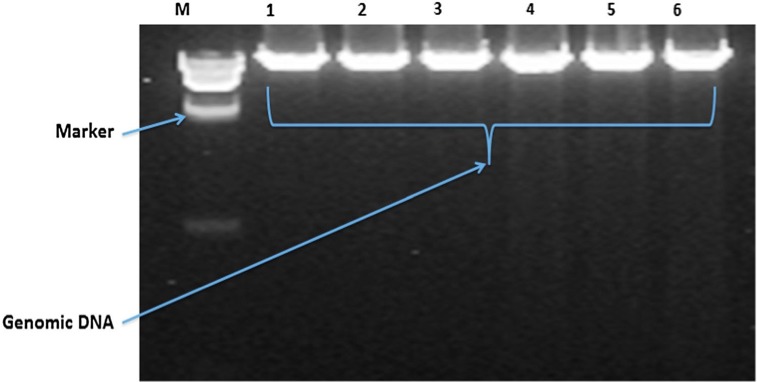
Gel picture of genomic DNA: **Lane 1**: 101PB; **2**: 102PB; **3**: 103PB; **4**: 104PB; **5**: 105PB; **6**: 106PB; M: Lambda/HindIII marker

**Plate 16:**
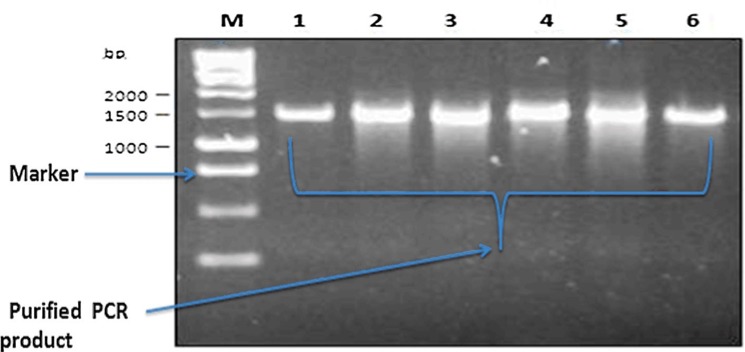
Gel picture of purified PCR product: **Lane 1**: 101PB; **2**: 102PB; **3**: 103PB; **4**: 104PB; **5**: 105PB; **6**: 106PB; M: 1 kb marker (Fermentas).

**Plate 17:**
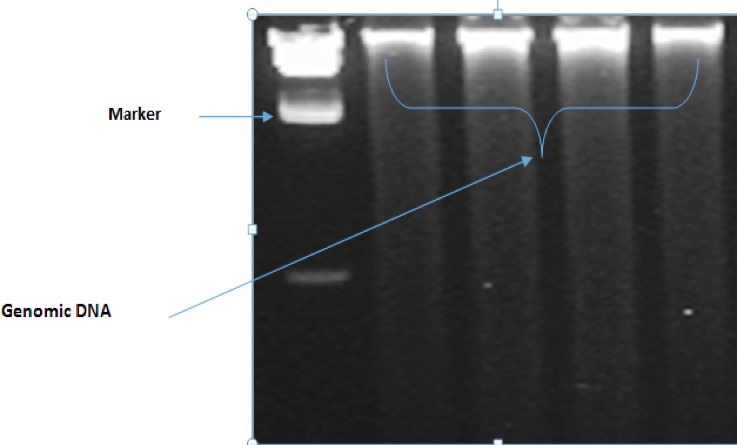
**Gel picture of genomic DNA: Lane 1**: 107 PF; **2:** 108 PF; **3:** 109 PF; **4:** 110 PF; **M:** Lambda/HindIII marker.

**Plate 18:**
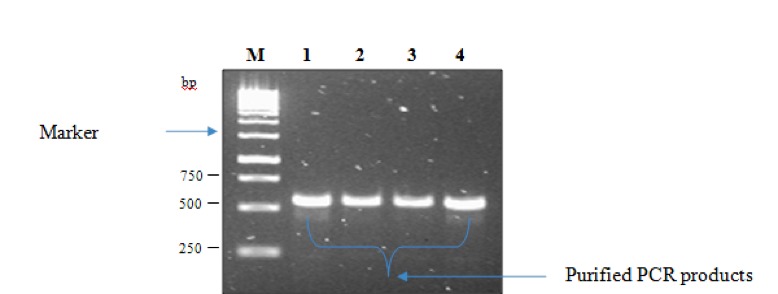
**Gel picture of purified PCR product: Lane 1**: 107 PF; **2:** 108 PF; **3:** 109 PF; **4:** 110 PF; **M:** 1 kb marker (Fermentas).

**Figure 1:**
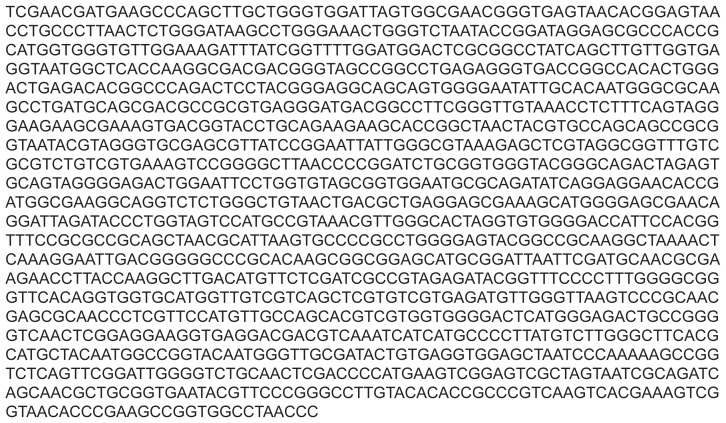
*Micrococcus luteus* 101 PB **(Accession NO. AB539843.1)**

**Figure 2:**
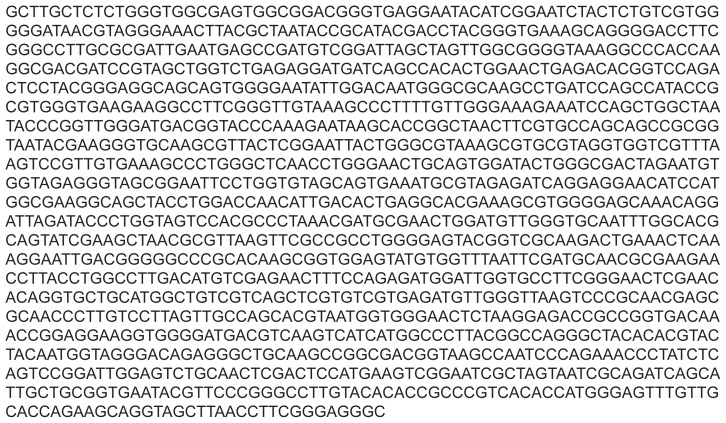
*Stenotrophomonas maltophilia* 102PB **(Accession No. JQ 619623.1)**

**Figure 3:**
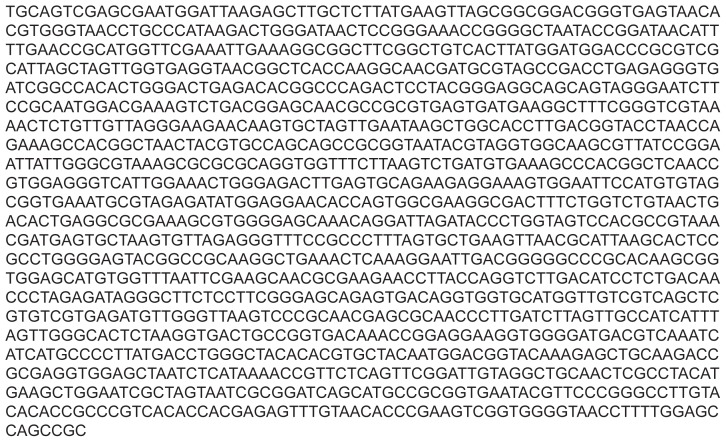
*Bacillus cereus* 103PB **(Accession No. JF 432000.1)**

**Figure 4:**
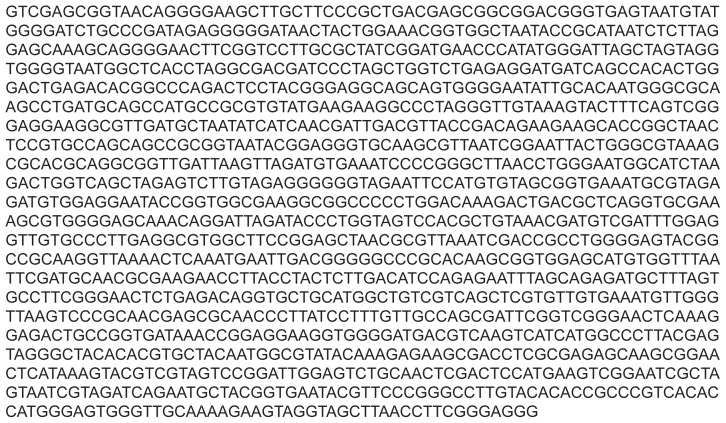
*Providencia vermicola* 104PB **(Accession No. KC775772.1)**

**Figure 5:**
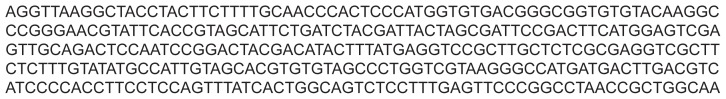

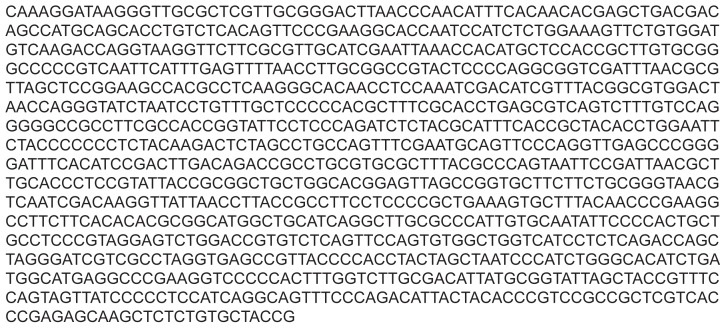
*Klebsiella pneumoniae* 105PB **(Accession No. GU128173.1)**

**Figure 6:**
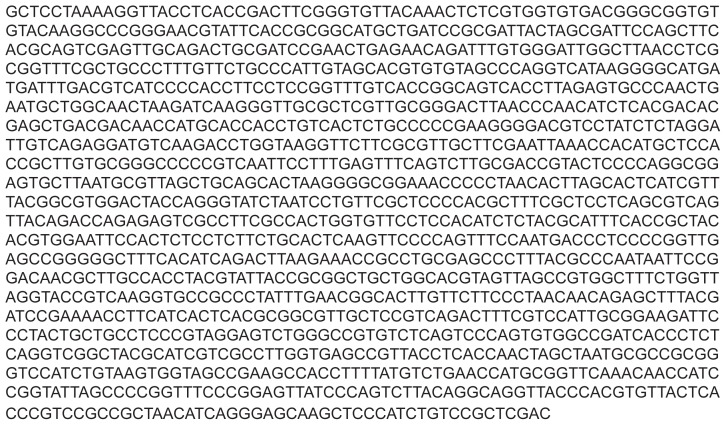
*Bacillus subtilis* 106PB (**Accession No. KF624694.1)**

**Figure 7:**
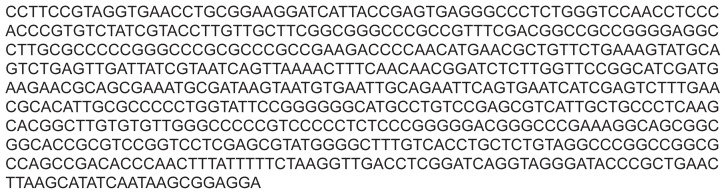
*Aspergillus fumigatus* 107PF **(Accession No. EU664467.1)**

**Figure 8:**
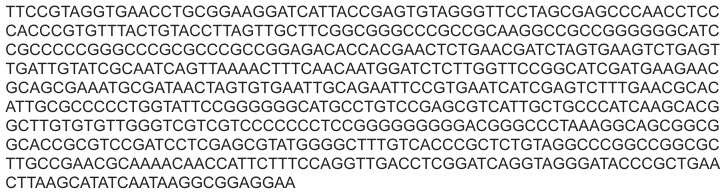
*Aspergillus nomius* 108PF **(Accession No. DQ467991.1)**

**Figure 9:**
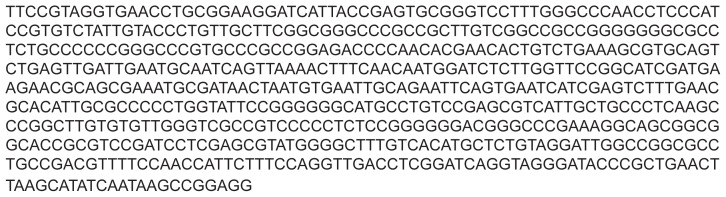
*Aspergillus niger* 109PF **(Accession No. KC119204.1)**

**Figure 10:**
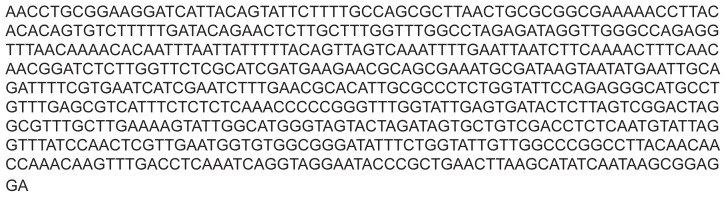
*Meyerozyma guilliermondii* 110PF **(Accession No. JN183444.1)**.

**Figure 11:**
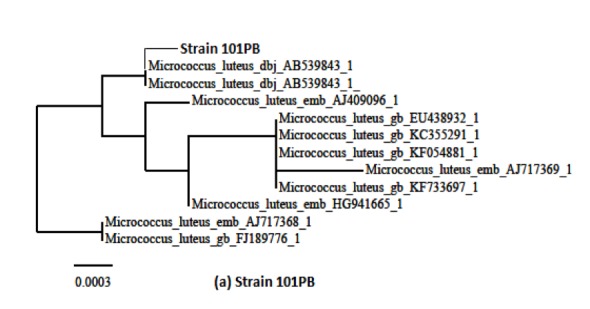
Phylogenetic tree of *Micrococcus luteus* 101PB based on 16S rRNA gene sequence comparisons

**Figure 12:**
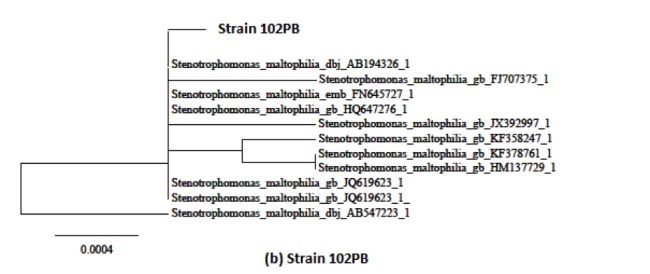
Phylogenetic tree of *Stenotrophomonas maltophilia* 102PB based on 16S rRNA gene sequence comparisons

**Figure 13:**
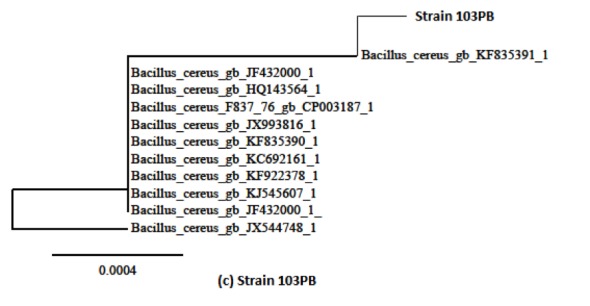
Phylogenetic tree of *Bacillus cereus* 103PB based on 16S rRNA gene sequence comparisons

**Figure 14:**
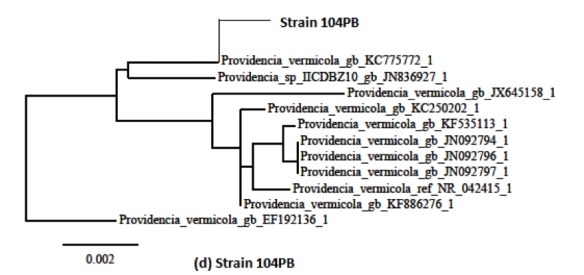
Phylogenetic tree of *Providencia vermicola* 104PB based on 16S rRNA gene sequence comparisons

**Figure 15:**
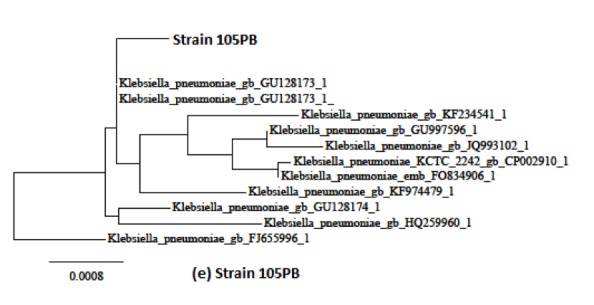
Phylogenetic tree of *Klebsiella pneumoniae* 105PB, based on 16S rRNA gene sequence comparisons

**Figure 16:**
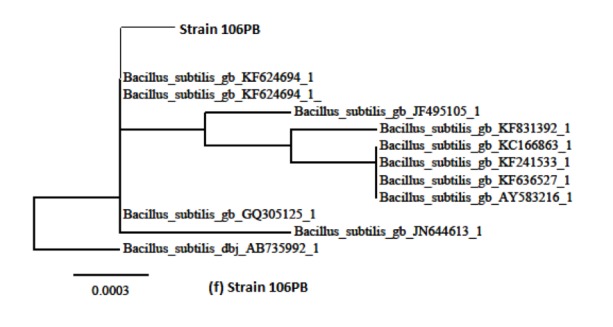
Phylogenetic tree of *Bacillus subtilis* 106PB based on 16S rRNA gene sequence comparisons

**Figure 17:**
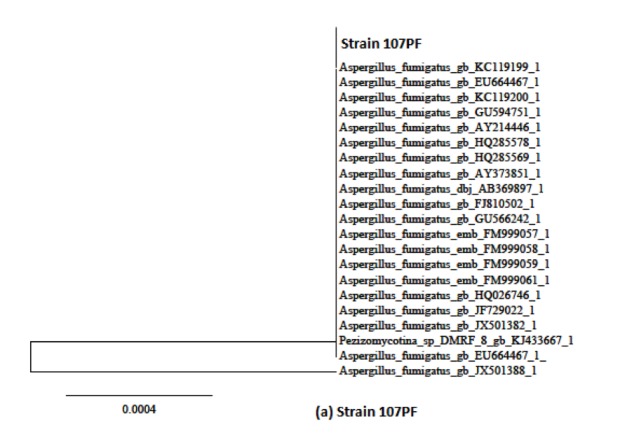
Phylogenetic tree of *Aspergillus fumigatus* 107PF based on 18S rRNA gene sequence comparisons

**Figure 18:**
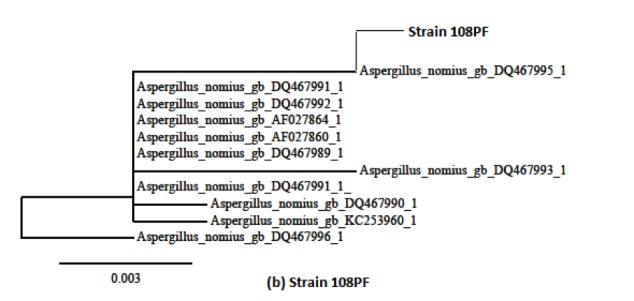
Phylogenetic tree of *Aspergillus nomius* 108PF based on 18S rRNA gene sequence comparisons

**Figure 19:**
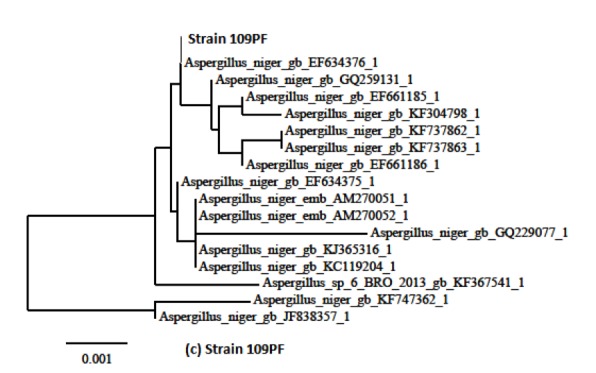
Phylogenetic tree of *Aspergillus niger* 109PF based on 18S rRNA gene sequence comparisons

**Figure 20:**
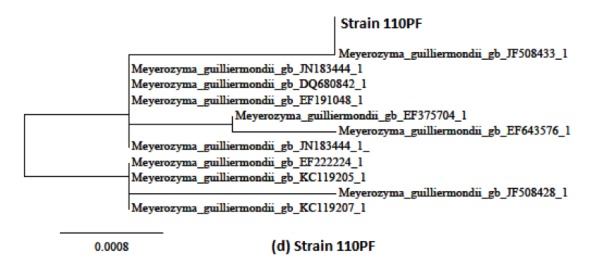
Phylogenetic tree of *Meyerozyma guilliermondii* 110PF based on 18S rRNA gene sequence comparisons

## APPENDIX A Identified bacteria in POME sample

**Table:**

Strains	Image	Gram’s reaction
*Micrococcus luteus* 101PB (Pure culture)	Plate 1	Gram positive cocci
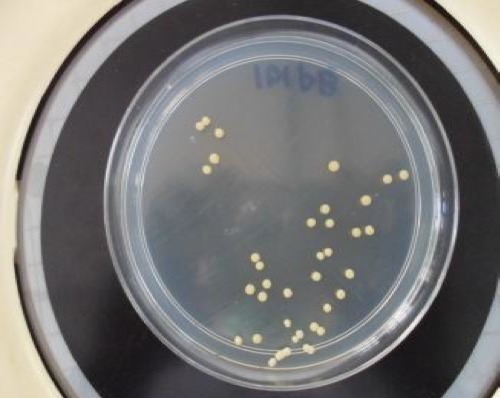
*Stenotrophomonas maltophilia* 102PB (Pure culture)	Plate 2	Gram negative rod
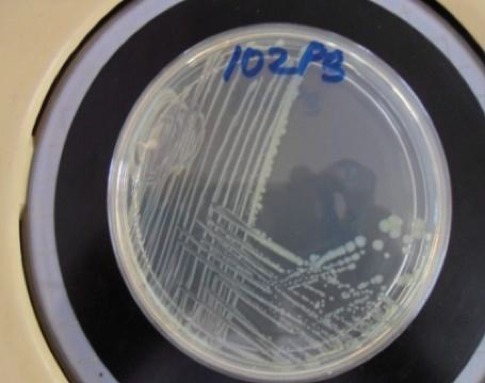
*Bacillus cereus* 103PB (Pure culture)	Plate 3	Gram positive rod
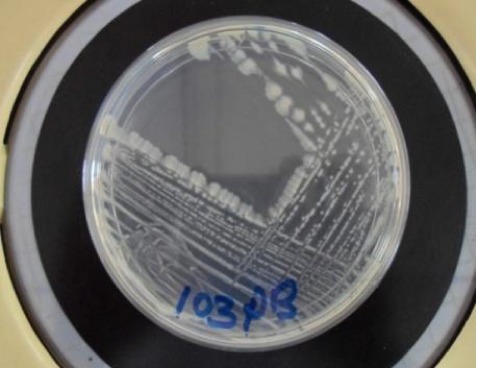
*Providencia vermicola* 104PB (Pure culture)	Plate 4	Gram negative rod
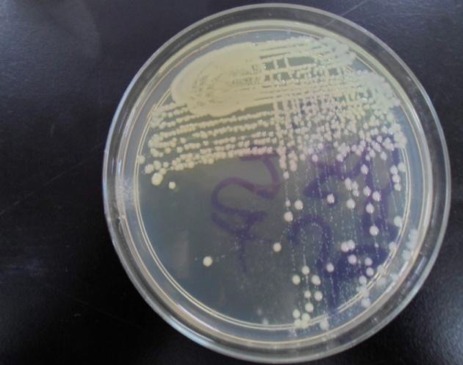
*Klebsiella pneumoniae* 105PB (Pure culture)	Plate 5	Gram negative rod
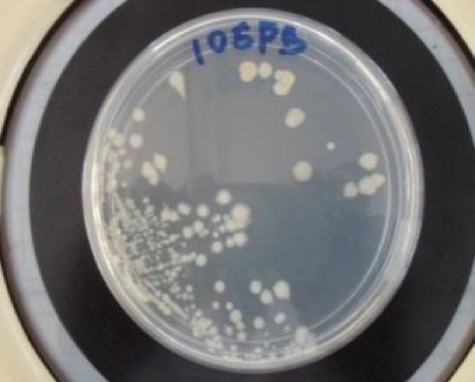
*Bacillus subtilis* 106PB. (Pure culture)	Plate 6	Gram positive rod
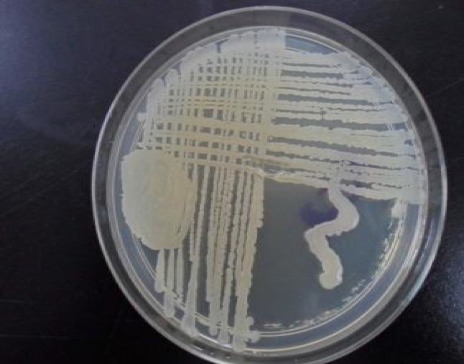

## APPENDIX B Identified fungi in POME sample

**Table:**

*Aspergillus fumigatus* 107PF (Microscopic staining)	Plate 7
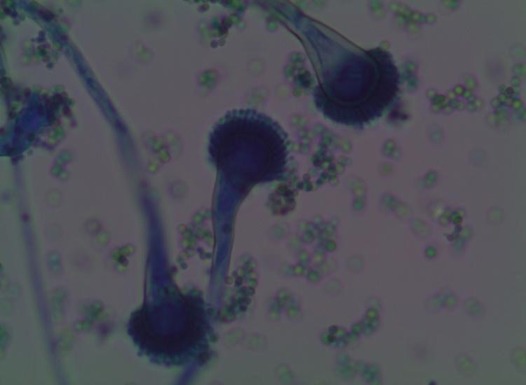
*Aspergillus nomius* 108PF (Microscopic staining)	Plate 8
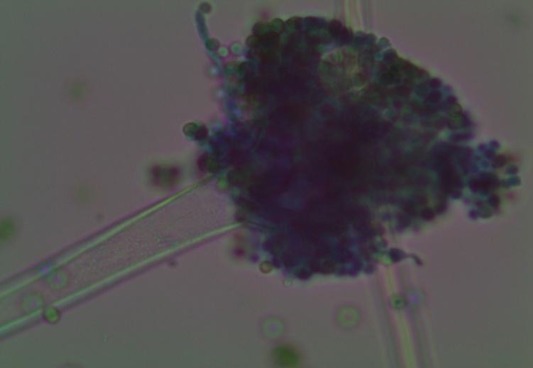
*Aspergillus niger* 109PF (Microscopic staining)	Plate 9
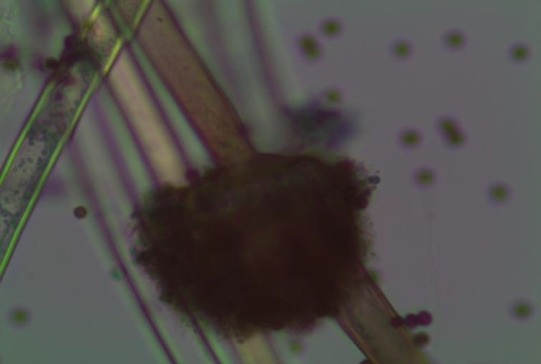
*Meyerozyma guilliermondii* 110PF (Microscopic staining)	Plate 10
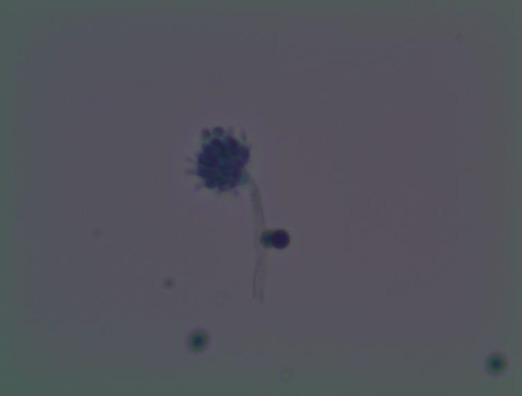
*Aspergillus fumigatus* 107PF (Pure culture)	Plate 11
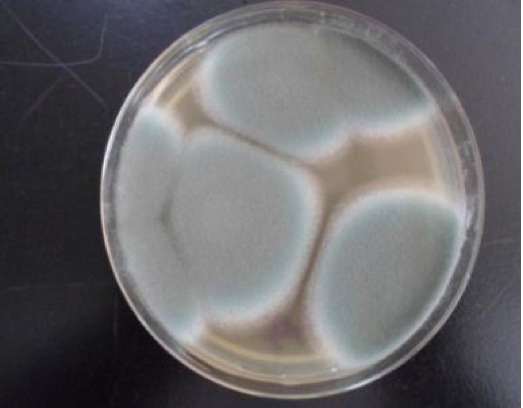
*Aspergillus nomius* 108PF (Pure culture)	Plate 12
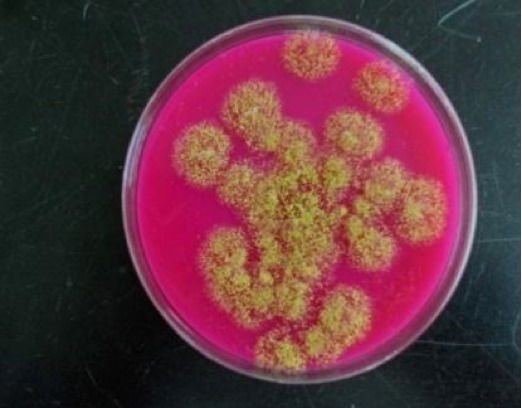
*Aspergillus niger* 109PF (Pure culture)	Plate 13
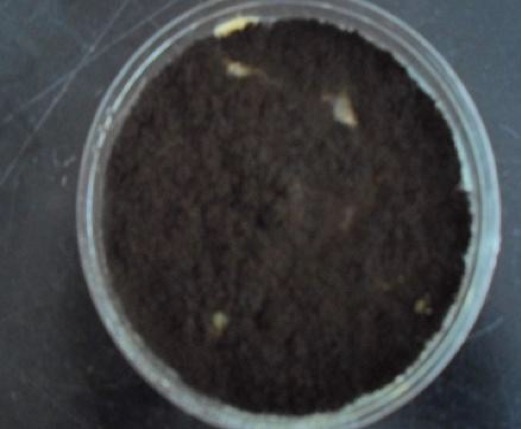
*Meyerozyma guilliermondii* 110PF (Pure culture)	Plate 14
